# Management of mycophenolate mofetil-induced acne in patients with Systemic Lupus Erythematosus: report of four cases and review of the literature

**DOI:** 10.31138/mjr.29.4.217

**Published:** 2018-12-18

**Authors:** Carlo Perricone, Fulvia Ceccarelli, Francesca Romana Spinelli, Simona Truglia, Roberta Priori, Guido Valesini, Fabrizio Conti

**Affiliations:** Lupus Clinic, Sezione di Reumatologia, Dipartimento di Medicina Interna, Sapienza Università di Roma, Rome, Italy

**Keywords:** Acne, Mycophenolate mofetil, Skin, Systemic Lupus Erythematosus

## Abstract

**Background:**

Mycophenolate mofetil (MMF) is an immunosuppressive drug currently used to treat Systemic Lupus Erythematosus (SLE). In addition to clinical efficacy, MMF use is also supported by a favorable profile of tolerance, with main side effects being nausea, diarrhea, headache, and, less frequently, leucopoenia. Acne is a relatively frequent adverse reaction to MMF that requires specific treatment and drug suspension.

**Investigations:**

Herein, we describe four cases of MMF-induced acne, none reporting past medical history of acne. The patients were diagnosed with SLE and lupus nephritis and treated with MMF. They developed papulo-pustular and nodular skin lesions consistent with acne. The lesions occasionally showed the appearance of macrocomedones or of unusual, nodular, oedematous lesions in gluteal region, or they had abscess-like features. Culture test demonstrated the presence of Staphylococcus Aureus. They resolved after MMF withdrawal and therapy with tetracycline and local pseudomonic-acid.

**Conclusions:**

Staphylococcus Aureus skin-localized infections inducing inflammatory/infectious acne may be a relatively frequent side-effect of MMF therapy in SLE. As soon as generalized, severe infections, due to Staphylococcus Aureus, may also occur in patients treated with MMF and even if antibiotics therapy is usually relatively effective, at least temporary MMF suspension may be suggested.

## INTRODUCTION

Mycophenolate mofetil (MMF) is an immunosuppressive drug the efficacy of which has been established in kidney, liver, or heart transplantation. It is converted to mycophenolic acid, and by inhibiting inosine monophosphate dehydrogenase, a lymphocyte specific enzyme, inhibits both T and B lymphocyte proliferation.^1^ In 1997 MMF (Cellcept®, Roche Pharma) was approved by the FDA to prevent renal allograft rejection, but over the last few years, its use in treating autoimmune disorders, mostly in Systemic Lupus Erythematosus (SLE),^2^ is becoming more widespread. Initially, MMF in SLE was used for diffuse proliferative lupus glomerulonephritis (World Health Organization [WHO] class-IV). Currently, MMF is also used to control general disease activity and other lupus manifestations including those haematological and cutaneous.^3^ In addition to clinical efficacy, MMF use is also supported by a favourable profile of tolerance, with main reported side effects being nausea, diarrhoea, headache, and, less frequently, leukopenia. Herein, we describe four cases of MMF-induced acne, none reporting past medical history of symptomatic acne and all of them caused by the presence of Staphylococcus Aureus. Resolution of the infection and MMF treatment suspension may be suggested in patients experiencing this adverse event.

## THE CASES

**Figure 1.**
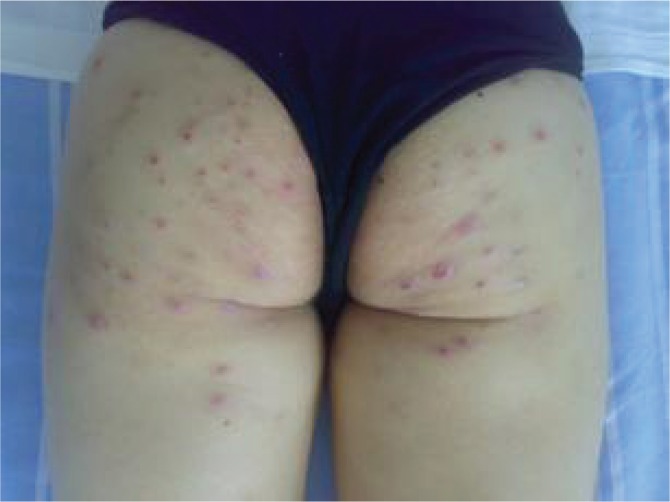
Papulo-pustules and nodules in gluteal region (Patient 1).

Patient 1, M.F., a 25-year-old woman, had been treated with prednisone, cyclosporin-A (CYA) and cyclophosphamide (CYC) for SLE since 1991 and for Lupus nephritis (WHO class-V) since 1999. For the poor control of proteinuria (2 g/24h) with glucocorticoid therapy, she started in 2002 MMF (2 g/daily) with normalization of proteinuria levels. In June 2008, the patient complained of papulopustules and nodules on the vulva and in gluteal region (*Figure 1*) with open and closed comedones. Contemporarily, paronychia and nasal furuncle appeared. Culture tests of two gluteal open comedones and of the nasal furuncle were undertaken and demonstrated the presence of Staphylococcus Aureus (SA) at the gluteal level. Thus, treatment with doxycycline 100 mg/daily together with local mupirocin in the form of 2% ointment applied three times per day was initiated while MMF was suspended producing clinical improvement.

Patient 2, F.C., a 33-years-old woman, had been treated since 1998 for SLE and retinal vasculitis using high doses-glucocorticoids, hydroxychloroquine, methotrexate, azathioprine and CYA. Due to several adverse events to such drugs and low benefit, MMF was introduced in November 2005 (2 g/daily) with efficacy on the clinical and laboratory picture. In March 2007, the patient complained of papulopustules and nodules in gluteal region and posterior region of the legs, starting with one single open comedone. Contemporarily, fever and generalized discomfort appeared, and the patient developed severe nodulocystic gluteal acne. Culture tests were undertaken at these regions together with a nasal culture test and demonstrated the presence of SA in the gluteal comedones. Thus, MMF treatment was suspended, while treatment with minocycline 100 mg/daily together with local mupirocin was initiated. Minocycline was interrupted after one week due to dizziness and substituted with Co-trimoxazole 320-1600 mg/daily. After two months, the resolution of the cutaneous lesions was observed.

Patient 3, K.C., a 33-year-old woman, diagnosed of SLE and Lupus nephritis (WHO class-IV-B) in 1998 was treated with CYC, and then with hydroxychloroquine, methotrexate, azathioprine and prednisone. Occasionally, proteinuria was still observed. In January 2006 due to the increase of proteinuria (> 1 g/24h), MMF was started at the dosage of 2 g/daily. After three months of therapy open and closed comedones in mammary, inguinal and gluteal region showing abscess-like features were noted. Culture tests of the comedones were undertaken together with a nasal culture test and SA was demonstrated in the gluteal comedones. Suspension of MMF and treatment with doxycycline 100 mg/daily together with topical mupirocin resulted in clinical improvement.

Patient 4, L.C., a 45-years-old woman, with a diagnosis of SLE and Lupus nephritis (WHO class-III-A), was treated with hydroxychloroquine and intravenous methylprednisone. For the persistence of positive urine protein analysis (> 1g/24h) she started in September 2008 MMF 3 g/daily with benefit on the renal disease. After one year of therapy open and closed comedones localized at the décolleté, arms and gluteal region were observed. Culture tests of the comedones localized at the arms were undertaken together with a nasal culture test. SA was present in both regions. Acne disappeared one month after MMF was suspended and treatment with doxycycline 100 mg/daily and topical mupirocin was started.

MMF was reintroduced in cases 2, 3 and 4 one month after the resolution of the event. The follow-up for cases 3 and 4 is up to two years with no other events. For case 2, MMF was continued till nowadays with clinical benefit and no further events.

## DISCUSSION

All the patients had a predominance of inflammatory/infectious acne lesions in which SA was isolated from the comedones. The clinical appearance differed from *acne vulgaris* and from monomorphic papules of corticosteroid-induced acne. Interestingly, the lesions were predominantly painful and occasionally showed the appearance of macrocomedones or of unusual, nodular, oedematous lesions in gluteal region, or abscess-like features. Moreover, the disappearance of the lesions after MMF withdrawal seems suggestive for a role of the drug in their induction. MMF-induced acne seems to be mainly due to immunosuppression and to an infection sustained by SA. Lesions were responsive to tetracycline and local pseudomonic acid together with the interruption of MMF therapy. The fact that SA was isolated in one nasal culture test may suggest that, at least in some cases, this is the source-site of the infection to be reclaimed. Of note, prednisone dosage was 7.5 mg/day in case 1 and 4; 5 mg/day in case 2 and 3 at the time of acne comparison. All the cases had reduced C4 and all but case 4 had reduced C3 at the time of the event. WBC count was within normal values in all cases; nonetheless, all the patients had haematological involvement (lymphopenia) in their clinical history.

No specific clinical reports of MMF-induced acne have been published yet. Side-effects associated with the immunosuppressive regimen can pose problems for patients with SLE. Skin lesions can be markedly distressing for patients reducing compliance to therapy. The mechanism of this MMF-induced acne is unknown, and the pathogenesis may include direct toxic effects or local immunosuppression. The longest-lasting experience with MMF has been accumulated in patients undergoing renal transplantation. In most of these cases MMF is used in combination with sirolimus to prevent allograft rejection. It has been widely reported that sirolimus frequently induces acne in a high number of patients. It may be supposed that a number of these cases may be due to MMF despite of sirolimus. Schaffellner et al. reported that 12/23 patients assuming sirolimus after renal transplantation showed dermatological side effects, six of which constituted by acne.^4^ Since 20/23 patients were also assuming MMF, it would be of interest to discriminate whether it was sirolimus or MMF to induce the skin lesions, not excluding the possibility of a synergistic effect. MMF side-effects are indeed well known and even various cutaneous lesions were anecdotally reported. The most common were infectious caused by mucocutaneous candidiasis, CMV syndrome, and Herpes Simplex.^5^ In a combination therapy with tacrolimus, MMF caused mouth ulceration. After MMF discontinuation, the lesions ameliorated.^6^ Oral ulcers were also reported by Naranjo et al., where authors concluded MMF was the suspicious drug.^7^ Indeed, another patient with the same combination therapy reported blisters on one hand and loose toenails; symptoms resolved after MMF therapy interruption. After the therapy with the drug was resumed, hand blisters and loose toenails recurred.^8^ Similarly, a patient with liver transplantation developed bullous eruption on hands and feet after MMF treatment. MMF was withdrawn and the cutaneous lesions resolved. After restarting therapy with MMF, the lesions recurred on the patient’s hand.^9^ All these reports sustain the hypothesis that MMF itself induces cutaneous lesions. MMF can reduce the expression of adhesion molecules on endothelial cells, leading to a decreased invasion of leukocytes in the target tissue, e.g. the skin.^10^ It may be postulated that specific immunosuppression at the skin level may predispose to localized infection. Indeed, MMF has been used for a variety of skin disorders included cutaneous manifestations of SLE, rationale being the reduction, directly induced by MMF, of leukocyte migration to skin.^11^ However, in patients affected with SLE, the sustaining immunological defect may lead into the development of SA infections; not only skin-localised, but also generalised. Indeed, not only could SA be isolated from the comedones of our patients, but also one patient affected with atopic dermatitis who developed SA septicaemia was already described in the literature.^12^ Unfortunately, we failed to find any clinical or laboratory feature that may predict the comparison of acne in MMF treated SLE patients.

In conclusion, acne may be a side-effect of MMF therapy in SLE. Infection with SA must be considered in these cases, and specific antibiotic therapy for reclaim of the germ is strongly suggested. Finally, even if therapy for acne is relatively effective, definitive treatment most often may rely on MMF suspension. It is noteworthy that reintroduction of MMF after SA eradication^13^ can be safe even in the long-term (up to 10 years of experience), even if the low number of patients evaluated cannot allow to draw definite conclusions.

## ABBREVIATIONS

CYC: Cyclophosphamide
MMF: Mycophenolate mofetil
SA: Staphylococcus Aureus
SLE: Systemic Lupus Erythematosus
